# Agricultural traits, silage quality and bacterial community structure changes of different sorghum varieties in cold regions of Hulunbuir

**DOI:** 10.1186/s12866-026-05437-2

**Published:** 2026-07-22

**Authors:** Zhaorui Han, Xun Chen, Sen Zong, Chao Jiang, Wenkai Zhu, Xiwen Su, Weiqiang Song, Xingzhao Zhou, Yanyan Liu, Wenge Li, Yulin Li, Meiling Hou, Yanzi Xiao

**Affiliations:** 1 College of Agriculture, Grass Industry Collaborative Innovation Research Center, Hulunbuir University, Hulunbuir, 021000 China; 2 College of Life Sciences, Baicheng Normal University, Baicheng, Jilin 137000 China; 3 Yangbo Animal Husbandry Development Service Co., Ltd., Evenk Banner, Hulunbuir, China

**Keywords:** Bacterial community, Cold region, Forage sorghum, Silage fermentation

## Abstract

Selecting high-quality forage varieties is essential for stabilizing regional forage supply and enhancing feed conversion efficiency. This study evaluated agronomic traits, nutritive value, and fermentation characteristics of five forage sorghum varieties (FS3501, QCDS, F10, 3701, and DJ) from the cold Hulunbuir region of China. All cultivars were harvested at the milk-ripe stage to measure plant height and dry matter (DM) yield. Forage was chopped, vacuum-sealed, and ensiled at 25 °C for 60 days. Post-fermentation, nutritional composition and fermentation parameters were analyzed. Additionally, 16 S rRNA gene sequencing with KEGG pathway analysis assessed bacterial community structure and metabolic function dynamics.Results showed that the 3701 (SB4) variety had superior production performance, with the highest (*p* < 0.05) plant height (2.54 m vs. 1.54–1.93 m) and DM yield (1567.65 kg/hm² vs. 800.40–1067.20 kg/hm²). SB4 also exhibited optimal silage fermentation, featuring the lowest pH (3.58 vs. 3.63–3.74), highest crude protein (6.79 vs. 3.44–6.43 g/kg DM), and lactic acid content (33.00 vs. 16.80–25.52 g/kg DM). Furthermore, SB4 significantly reduced neutral detergent fiber (NDF) and acid detergent fiber (ADF) levels. Microbial analysis revealed decreased α-diversity and a shift from Proteobacteria to Firmicutes, with SB4 silage dominated by *Lactobacillus*. KEGG functional prediction indicated that carbohydrate metabolism was the predominant metabolic pathway. These findings highlight SB4 as a superior forage variety suitable for cultivation and silage production in high-latitude cold regions like Hulunbuir.

## Introduction

Sorghum (*Sorghum bicolor* (L.) Moench), a globally distributed gramineous crop, plays a pivotal role in ensuring forage supply and feed security for the livestock industry [1]. Currently cultivated across diverse climatic zones, sorghum has emerged as an ideal candidate for addressing the deficit in high-quality roughage due to its exceptional biomass yield, superior abiotic stress resistance, and relatively short growth cycle [2]. Characterized by tender stems and leaves, high palatability, and significant dry matter digestibility, sorghum provides abundant biomass and exhibits excellent conversion efficiency during anaerobic fermentation, making it a premier raw material for high-quality silage production.

In the context of high-quality livestock development, variety screening is directly linked to the stability of regional forage supply and the enhancement of feed conversion efficiency [3]. Numerous global studies have demonstrated that yields and silage quality fluctuate significantly among varieties in the same environment, driven by genetic variations in agronomic traits, cell wall compositions, and epiphytic bacterial community structures [4]. Therefore, a critical objective in variety screening is to identify cultivars with high biomass accumulation and excellent ensiling characteristics, which can facilitate the rapid initiation of lactic acid bacteria-led fermentation, thereby preserving nutrients and inhibiting pathogenic microorganisms. Therefore, the selection and promotion of highly adaptable sorghum varieties can optimize the biomass attributes of feedstocks, providing a critical material guarantee for the high-quality transformation of regional animal husbandry.

The Hulunbuir cold region, a vital livestock production base in China, currently faces severe challenges. This region is characterized by a short growing season, uneven seasonal precipitation, and prolonged, frigid winters [5]. A defining feature of local animal husbandry is the seasonal nutritional imbalance: while pastures are lush in summer, the dormant period—lasting over seven months—subjects livestock to a recurring cycle of “fat in summer, strong in autumn, thin in winter and dying in spring” [6]. Furthermore, there is a heavy reliance on expensive external commercial concentrates during winter. Structural feed shortages and seasonal nutritional deficits have become the primary bottlenecks hindering livestock productivity and the growth of pastoralists incomes. Consequently, screening for optimal sorghum varieties in the Hulunbuir cold region is of profound strategic importance. Due to the extreme temperature fluctuations during the harvest period in cold seasons, conventional varieties often suffer from delayed fermentation initiation, substantial dry matter loss, and susceptibility to mold. It is imperative to identify varieties that possess robust low-temperature adaptability, rapid biomass accumulation within limited accumulated heat units, and “fast acidification” characteristics. The selection and dissemination of superior sorghum varieties adapted to high-latitude frigid climates will not only address the local shortage of high-quality silage but also enhance the sustainability of livestock production in high-latitude ecosystems, providing scientific support for securing over-wintering forage supplies in cold regions.

## Materials and methods

### Substrate and silage preparation

The field experiment was conducted in 2024 at the Experimental Farm of Hulunbuir University, Yakeshi Campus, Inner Mongolia, China (120°28′E to 122°29′E, 47°39′N to 50°21′N), situated at an elevation of 600–700 m. The region experiences a continental cold-temperate climate with an annual average temperature ranging from − 2.5 °C to 0.5 °C and an extreme minimum of -45 °C. The growing season is characterized by significant diurnal temperature fluctuations and a limited thermal resource, with an annual effective accumulative temperature (≥ 10 °C) of 1,700–2,100 °C. Annual precipitation is approximately 388.70–477.90 mm, with over 70% concentrated between June and August, while the average annual relative humidity remains between 65% and 70%. The soil at the site is classified as chernozem, characterized by a loose texture, a pH of approximately 7.0, and moderate fertility with high organic matter content. Five forage sorghum (Sorghum bicolor) cultivars, namely FS3501, QCDS, F10, 3701, and DJ (Beijing Zhengdao Seed Industry Co., Ltd.), were selected for the study and designated as SB1, SB2, SB3, SB4, and SB5, respectively. These cultivars were chosen for their proven cold tolerance, rapid biomass accumulation, and high crude protein content, demonstrating superior adaptability to the high-latitude, frigid environments typical of the Hulunbuir region.

A randomized complete block design was employed, with five cultivars planted in three replicates each, totaling 15 plots. Each plot covered an area of 20 m^2^ (4 m×5 m), with protective rows established around the experimental field to minimize edge effects. Sowing was performed on May 17, 2024, using the hole-sowing method (2–3 seeds per hole) at a depth of 5 cm, with a plant spacing of 20 cm and row spacing of 40 cm. Prior to sowing, a compound fertilizer (N: P_2_O_5_:K_2_O = 12:18:15) was applied at a rate of 300 kg/ha (equivalent to 20 kg/hm²).

All cultivars were harvested uniformly at the milk-ripe stage on September 9, 2024, and the harvested material was directly ensiled without additives following a completely randomized design with three replicates per treatment. Approximately 500 g of chopped forage from each sample was packed into specialized polyethylene vacuum bags (25 cm × 35 cm, 0.12 mm thickness; Dalian Chengwei Plastic Products Co., Ltd., China). The bags were vacuum-sealed using a DZ-400 vacuum packaging machine (Wenzhou Zhengtai Packing Machinery Co., Ltd., China) to establish anaerobic conditions and subsequently stored in a cool, dark indoor environment for fermentation at 22–28 °C (average approximately 25 °C) for 60 days.

After 60 days of anaerobic fermentation, the silage bags were opened for sampling. A representative sub-sample of approximately 200 g was collected using the quartering method. The samples were immediately lyophilized using an Alpha 1–2 LDplus freeze-dryer (Martin Christ GmbH, Osterode am Harz, Germany). The dehydrated samples were then pulverized using an FW100 mini-grinder (Tianjin Taisite Instrument Co., Ltd., China) and passed through a 40-mesh (0.43 mm) sieve. The resulting fine powder was stored for subsequent analysis of chemical composition and fermentation parameters.

### Production performance analysis

To minimize potential spatial heterogeneity caused by edge effects and uneven light distribution within plots, border plants were excluded and all measurements were conducted within the interior sampling area of each plot. During harvesting, three representative plants were randomly selected from different positions within the central area of each plot. Plant height was measured as the distance from the ground surface to the highest point of the upright leaves and averaged across the selected plants [7]. For each plot, three individual plants were selected to determine leaf weight, stem weight, ear length, and leaf number of the first basal internode. After harvest, three 1 m × 1 m quadrats were randomly established within the interior area of each plot for dry matter yield determination [8].

### Physicochemical indicators

Fresh forage sorghum cultivars and silage samples after 60 days of fermentation were dried in a forced-air oven at 65 °C for 48 h to determine DM content. The dried samples were ground to pass a 1 mm sieve using a high-speed pulverizer (FW100, Tianjin Taisite Instrument Co., Ltd., Tianjin, China) and subsequently analyzed for chemical composition [9]. NDF and ADF contents were determined according to the method described by Van Soest [10]. Ammonia nitrogen (NH₃-N) content was measured as described by BRODERICK G [11]. The moisture contents of five fresh forage sorghum varieties before ensiling were as follows: SB1 at 65.68%, SB2 at 64.75%, SB3 at 66.36%, SB4 at 65.79%, and SB5 at 66.64%.

Ten grams of silage samples were immediately immersed in 90 mL of sterile water to collect microorganisms, followed by shaking at 160 rpm for 2 h at 4 °C to obtain the filtration. Organic acid levels in silage filtrates were determined by HPLC with a UV detector set at 210 nm. The mobile phase was a 3 mmol/L HClO_4_ solution at a flow rate of 1.00 mL·min^-1^, and column temperature was maintained at 50 °C.

### Bacterial community analysis

The mixture was filtered through two layers of sterile gauze, and the residue was rinsed several times with sterile water to recover any residual microorganisms. The filtrates were centrifuged at 10,000 × g at 4 °C for 15 min, and the bacterial pellets were stored at -80 °C. Total bacterial DNA was extracted using the TIANamp Bacteria DNA Isolation Kit (DP302-02, Tiangen, Beijing, China) according to the manufacturer’s instructions. The extracted DNA was subsequently used to amplify the V3-V4 hypervariable region of the 16 S ribosomal RNA (rRNA) gene using universal primers (forward: 5′-ACTCCTACGGGAGGCAGCA-3′; reverse: 5′-GGACTACHVGGGTWTCTAAT-3′). Polymerase chain reaction (PCR) amplification was performed following previously described methods [12]. The diversity indices and bacterial community structures of forage sorghum cultivars silage from different cultivars were analyzed using the Weikemeng Biocloud Platform (https://www.bioincloud.tech/, accessed on Nov 25, 2025).

It should be noted that due to the current version of the reference database provided by the analysis platform, the genus *Lactobacillus* in this study is referred to in its broad sense (sensu lato), retaining the classic nomenclature prior to its recent taxonomic reclassification.

### Statistical analysis

All results are presented as the mean values of three replicates. Nutritional and fermentation data were analyzed using single-factor analysis of variance with SPSS software (version 27.0; IBM Corp., Armonk, NY, USA) [13]. Bacterial data were analyzed using the Basebio online platform(https://www.omicshare.com/tools/home/report/reportvenn.html). Bacterial functional analyses were conducted using Origin (OriginLab Corporation, Northampton, MA, USA) software. Correlation analysis was performed to determine the relationships between bacterial taxonomic characteristics and silage quality parameters by calculating Spearman correlation coefficients, and the results were visualized using an online analysis tool (https://chiplot.online/) [14]. Statistical significance was defined at a probability level of 0.05.

## Results

### Comparative analysis of plant height and hay yield

The yield characteristics of different varieties of forage sorghum are shown in the table (Table 1). Significant (*p* < 0.05) differences were observed among cultivars in terms of both hay yield and plant height. Specifically, the cultivar SB4 exhibited the highest hay yield (1567.65 kg/hm² vs. 800.40–1067.20 kg/hm²), which was significantly greater (*p* < 0.05) than that of other cultivars. In contrast, SB3 recorded the lowest yield (800.40 kg/hm² vs. 900.45–1567.65 kg/hm²). Regarding plant height, SB4 was the tallest (2.54 m vs. 1.54–1.93 m), significantly (*p* < 0.05) surpassing all other cultivars, while SB1 had the shortest height (1.54 m vs. 1.83–1.93 m).

**Table 1:** Growth performance of different varieties of sorghum for feed

Items	Forage sorghum cultivar					SEM	*P*-value
	SB1	SB2	SB3	SB4	SB5		
Dry matter yield (kg/hm^2^)	920.46 ± 26.68^bc^	1067.20 ± 106.76^b^	800.40 ± 53.36^c^	1567.65 ± 40.02^a^	900.45 ± 20.01^bc^	28.501	< 0.001
Plant height (m)	1.54 ± 0.01^c^	1.83 ± 0.02^b^	1.93 ± 0.06^b^	2.54 ± 0.08^a^	1.86 ± 0.03^b^	0.040	< 0.001

^1^*SEM* Standard error of the mean. SB1, FS3501; SB2, QCDS; SB3, F10; SB4, 3701; SB5, DJ

^2^a-c indicating significant differences at *p* < 0.05 level

### Chemical composition and fermentation characteristics of silage

The pH values of all cultivated varieties decreased significantly, with the pH value of the SB4 group (3.58 vs. 3.63–3.74) being markedly lower (*p* < 0.05) than that of the other groups (Table 2). In this study, LA (33.00 g/kg DM vs. 16.80–25.52 g/kg DM) concentrations in SB4 were significantly higher (*p* < 0.05) than those in the other cultivars. The CP content in SB4 was significantly higher (*p* < 0.05) than that in other groups (6.79 g/kg DM vs. 3.44–6.43 g/kg DM). The NDF content of SB4 was lower than that of SB5 (539.24 g/kg DM vs. 558.36 g/kg DM), the ADF content was significantly lower (*p* < 0.05) than that of other varieties (338.11 g/kg DM vs. 334.91–346.44 g/kg DM).

**Table 2:** Nutritional composition and fermentation quality of different forage sorghum varieties

Items	Forage sorghum cultivar					SEM	*P*-value
	SB1	SB2	SB3	SB4	SB5		
pH	3.70 ± 0.01^ab^	3.63 ± 0.01^bc^	3.74 ± 0.05^a^	3.58 ± 0.02^c^	3.74 ± 0.01^a^	0.020	0.002
Lactic acid (g/kg DM)	22.41 ± 0.66^ab^	23.83 ± 0.60^ab^	25.52 ± 0.78^ab^	33.00 ± 1.28^a^	16.80 ± 0.22^b^	0.708	0.015
Acetic acid (g/kg DM)	4.32 ± 0.01^b^	5.91 ± 0.06^ab^	7.73 ± 0.03^a^	6.85 ± 0.07^a^	6.12 ± 0.07^ab^	0.048	0.014
NH_3_-N (g/kg TN)	7.33 ± 0.33^a^	6.00 ± 0.58^ab^	5.33 ± 0.33^b^	5.67 ± 0.33^b^	5.67 ± 0.33^b^	0.380	0.036
Dry matter (%FW)	34.32 ± 0.14^ab^	35.25 ± 0.06^a^	33.64 ± 0.18^b^	34.21 ± 0.16^ab^	33.36 ± 0.13^b^	0.134	0.016
Crude protein (g/kg DM)	3.44 ± 0.19^c^	4.99 ± 0.17^b^	5.40 ± 0.22^b^	6.79 ± 0.10^a^	6.43 ± 0.36^a^	0.328	< 0.001
Neutral detergent fiber (g/kg DM)	485.71 ± 12.72^b^	518.53 ± 15.21^ab^	526.44 ± 13.33^ab^	539.24 ± 12.62^a^	558.36 ± 6.50^a^	17.960	0.022
Acid detergent fiber (g/kg DM)	325.50 ± 3.12^b^	334.91 ± 4.12^ab^	348.95 ± 6.05^a^	338.11 ± 4.32^ab^	346.44 ± 6.06^a^	4.734	0.040

^1^*SEM* Standard error of the mean. *DM* Dry matter, *FW* Fresh weight, *TN* Total nitrogen. SB1, FS3501; SB2, QCDS; SB3, F10; SB4, 3701; SB5, DJ

^2^a-c indicating significant differences at *p* < 0.05 level

### Analysis of bacterial communities in silage of different forage sorghum cultivars

High-throughput sequencing analysis of the 16 S rRNA gene revealed significant (*p* < 0.05) differences in the bacterial community structures among the treatment groups (SB1, SB2, SB3, SB4, and SB5) during the ensiling of forage sorghum.

**Fig. 1:**
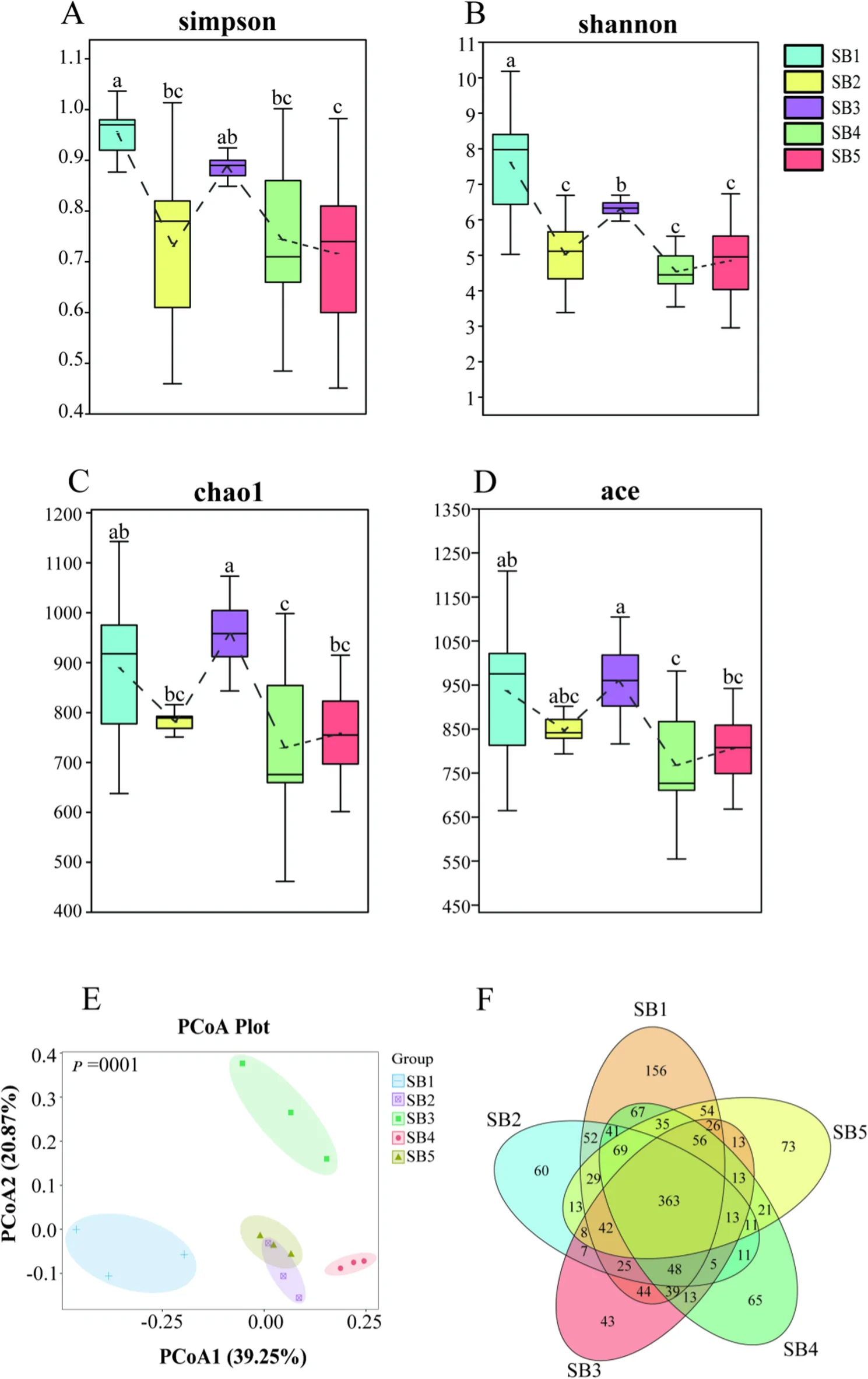
Impact of different cultivars on the microbial community structure of forage sorghum silage. **A**, **B**, **C**, and **D** illustrate the differences in the Simpson, Shannon, Chao1, and ACE indices among the groups, respectively. Panel (**E**) presents the PCoA based on Bray–Curtis distances, illustrating compositional differences among treatments. Panel (**F**) shows the Venn diagram, highlighting the shared and unique OTUs between fresh material and silage. (SB1: FS3501, SB2: QCDS, SB3: F10, SB4: 3701, SB5: DJ)

Alpha diversity shows that the significant decrease in Shannon and Chao1 (Fig. 1B and C) indices after ensiling indicate that the anaerobic and acidic environment acted as a strong environmental filter, selecting for a few specialized, acid-tolerant bacterial taxa (primarily within Firmicutes), which is a hallmark of successful silage fermentation. The Simpson index of the SB4 group (Fig. 1A) was significantly lower than that of the other groups, while its ACE indices (Fig. 1D) were the lowest (*p* < 0.05) among all treatments.

Principal Coordinate Analysis (PCoA) revealed a distinct separation in bacterial community structures among the different groups (Fig. 1E). The first two principal coordinates, PCoA1 and PCoA2, explained 39.25% and 25.87% of the total variance, respectively. Additionally, the Venn diagram (Fig. 1F) showed that the number of operational taxonomic units (OTUs) in SB1, SB2, SB3, SB4, and SB5 was 156, 60, 43, 65, and 73, respectively.

**Fig. 2:**
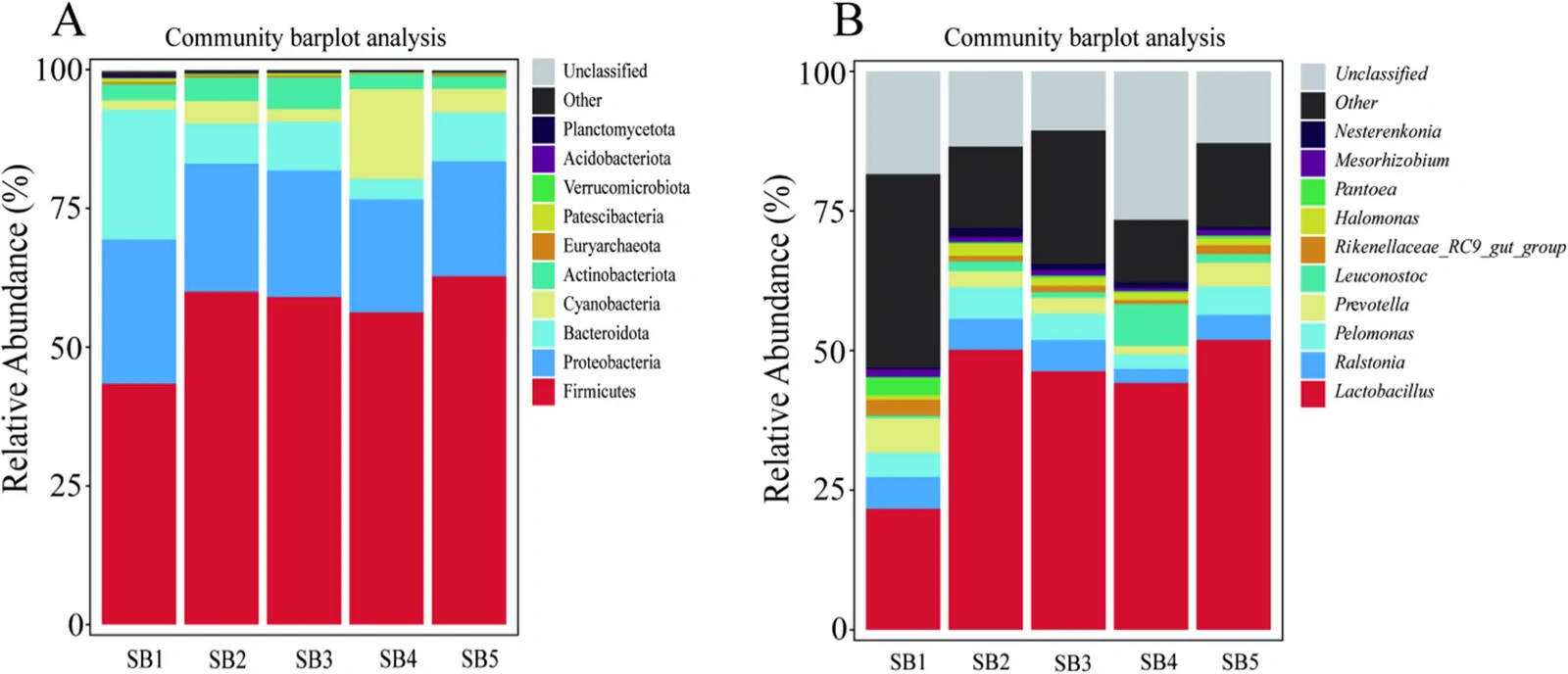
Relative abundance of microbial taxa in forage sorghum silage across different cultivars (SB1-SB5) after 60 days of fermentation, determined via 16 S rRNA gene sequencing. The bar plots illustrate shifts in the bacterial community structure at the phylum level (**A**) and the genus level (**B**)

At the phylum level (Fig. 2A), Firmicutes dominated the SB5 group, accounting for 60.1% of the total sequences. Throughout the fermentation process, the bacterial communities across all groups exhibited a directional successional shift from Proteobacteria to Firmicutes. This evolution in community structure indicates the successful transition of the fermentation system from an aerobic state to an anaerobic lactic acid fermentation phase. At the genus level (Fig. 2B), *Lactobacillus* exhibited the highest relative abundance, which was significantly higher (*p* < 0.05) in the SB5 group compared to the other groups. As the core functional genus,* Lactobacillus* can efficiently utilize available fermentable substrates to rapidly reduce the environmental pH.

**Fig. 3:**
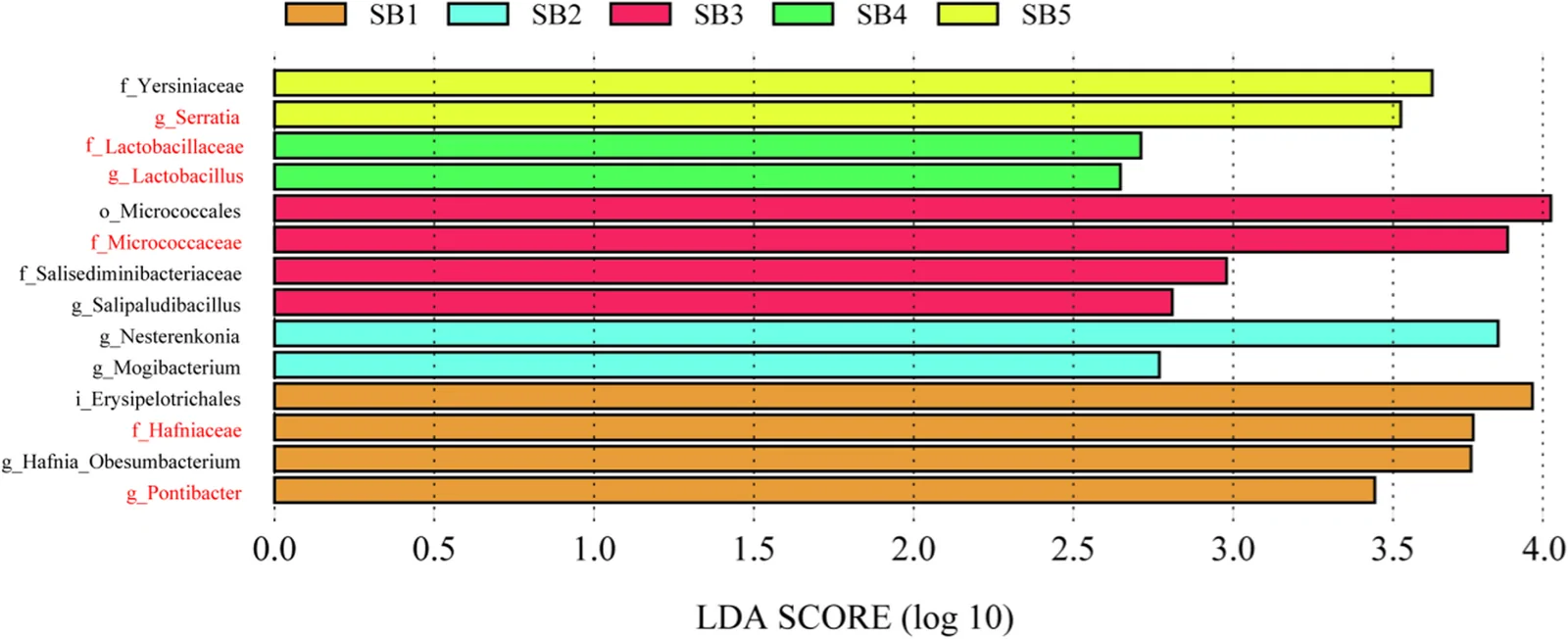
Identification of statistically significant microbial biomarkers in the silage of different forage sorghum cultivars using Linear discriminant analysis Effect Size (LEfSe). The bar chart displays taxa with significant differential abundance across the five cultivar groups (SB1 to SB5) after 60 days of ensiling. Taxa marked in red represent key phyla, families, and genera driving the community differences

LEfSe analysis (Fig. 3) showed that by the end of the fermentation process, the SB1 variety was enriched with Hafniaceae, the SB2 variety with Nesterenkonia, the SB3 variety with Micrococcaceae, the SB4 variety was enriched *Lactobacillus*, and the SB5 variety was enriched with *Serratia*.

### Pearson correlation of physicochemical and microbiological indices

**Fig. 4:**
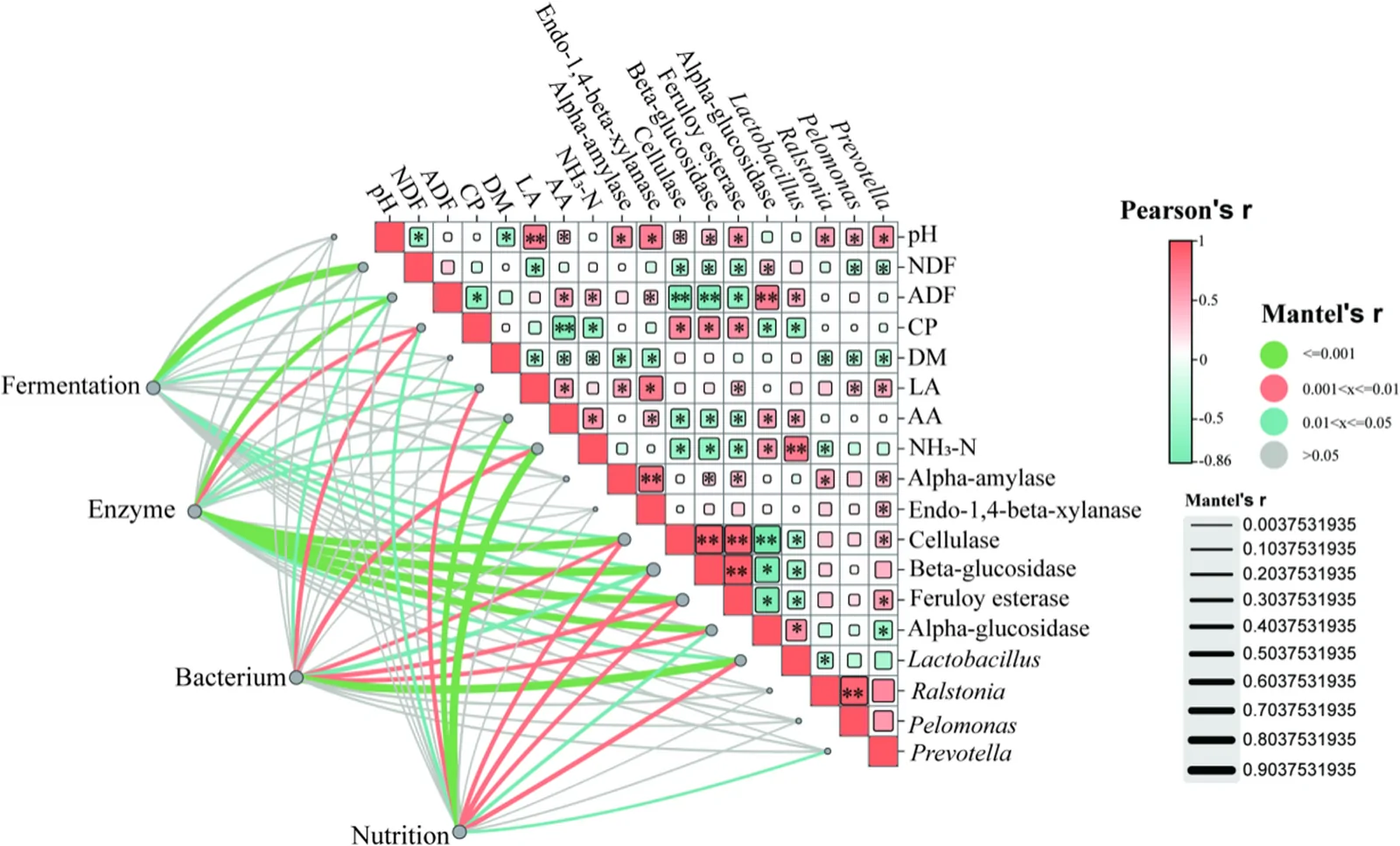
Mantel analysis of nutrient composition, fermentation parameters, enzyme indices, and bacterial communities among different forage sorghum varieties. Line color indicates the direction of correlation: red represents a positive correlation and green represents a negative correlation. Line thickness is proportional to the magnitude of the Mantel correlation coefficient (r), with thicker lines indicating a stronger correlation and thinner lines indicating a weaker correlation. Asterisks on lines denote the statistical significance of the correlation (*P < 0.05, **P < 0.01); nonsignificant correlations (*P* > 0.05) are not displayed. PH, pH value; NDF, Neutral detergent fiber; ADF, Acid detergent fiber; CP, Crude protein; DM, Dry matter; LA, Lactic acid; AA, Acetic acid; NH₃-N, Ammonia nitrogen

Pearson correlation analysis revealed (Fig. 4) a highly significant (*p* < 0.01) negative correlation between pH value and LA content. Among the nutritional components, CP exhibited a significant positive correlation with ammonia nitrogen (NH_3_-N), while NDF showed a highly significant (*p* < 0.01) positive correlation with pH. Cellulase and endo-1,4-beta-xylanase displayed highly significant (*p* < 0.01) positive correlations with LA content, and significant negative correlations with pH value. Additionally, *Lactobacillus* was positively correlated with LA content and negatively correlated with pH value, both at a significant level. *Ralstonia* showed a significant positive correlation with NH_3_-N, and Prevotella exhibited a significant positive correlation with AA.

### Functional prediction of bacterial communities based on KEGG pathways

**Fig. 5:**
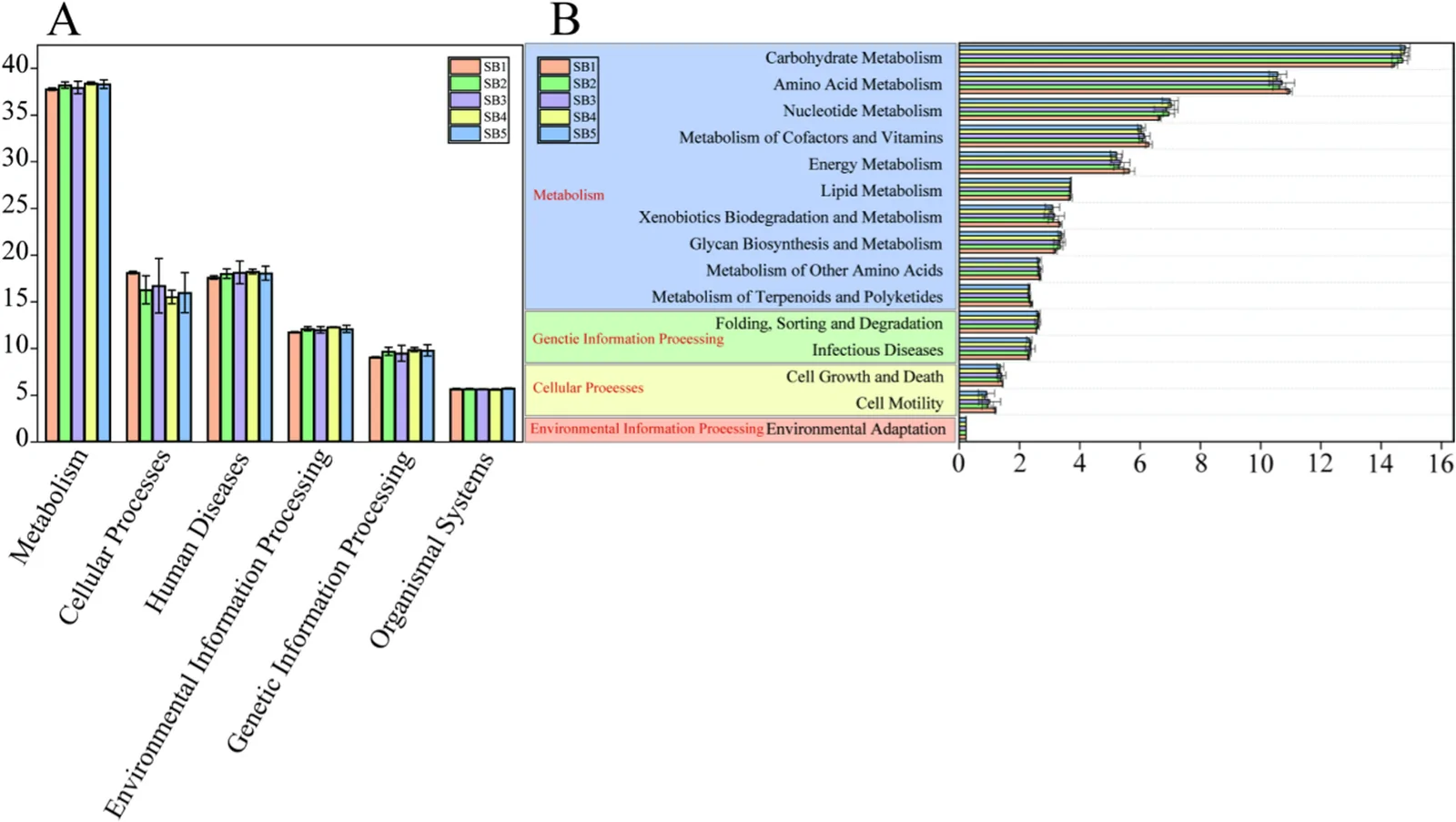
Functional prediction of microbial communities based on KEGG pathways. **A** Level 1 metabolic pathways. **B** Level 2 KEGG ortholog functional predictions showing the relative abundances of the top 15 metabolic functions

At KEGG level 1 (Fig. 5A), metabolism was the most predominant category across all five cultivars, with a relative abundance ranging from approximately 37% to 39%. This was followed by “Cellular Processes”, “Human Diseases”, and “Environmental Information Processing”. At KEGG level 2 (Fig. 5B), within the “Metabolism” category, Carbohydrate Metabolism was the most abundant functional pathway (14–15%), followed by Amino Acid Metabolism (10.50%). Other pathways, such as Nucleotide Metabolism and Energy Metabolism, also maintained consistent levels across SB1 to SB5.

### KEGG-Based prediction of enzymatic functions in the bacterial community

**Fig. 6:**
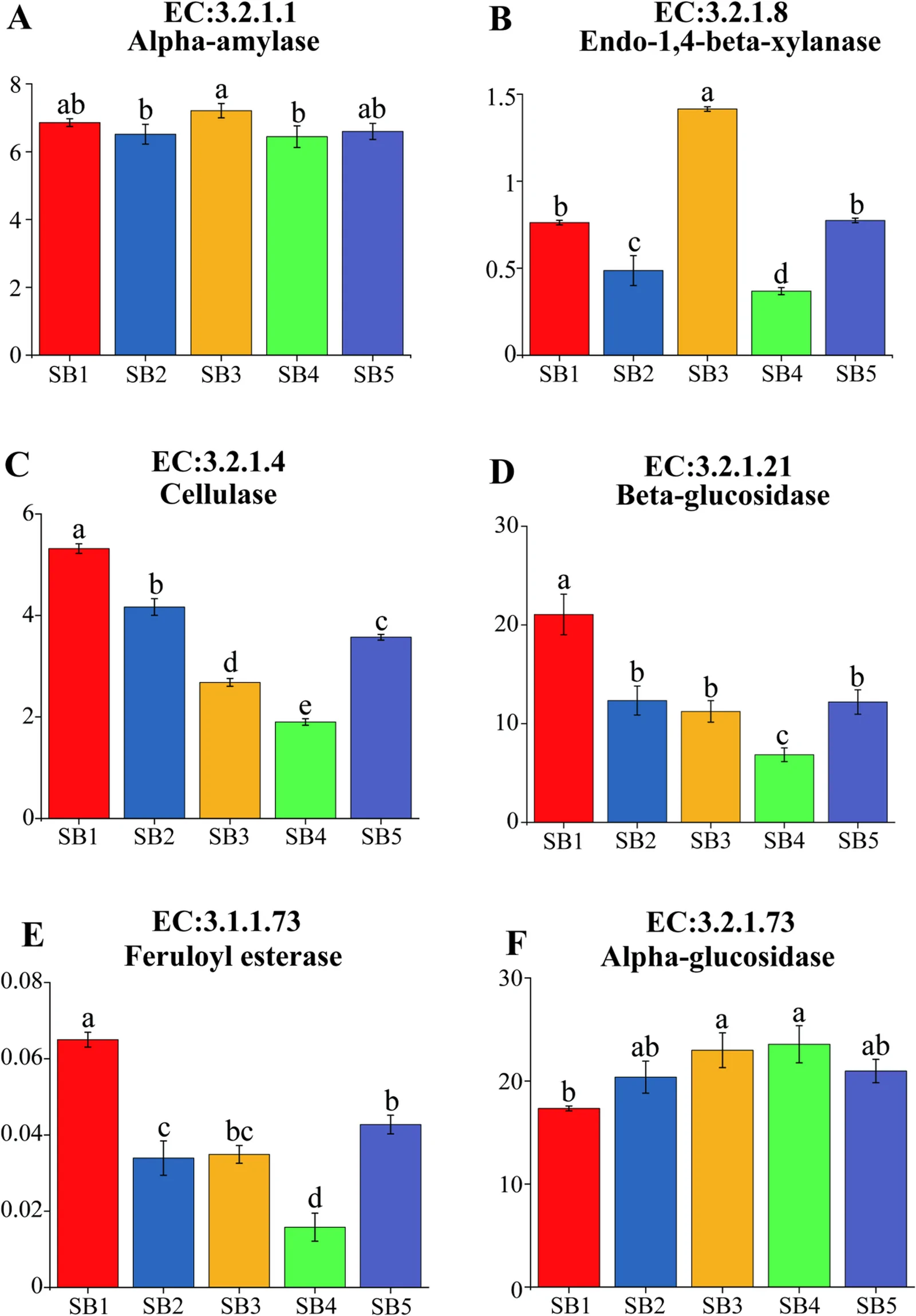
Changes in key enzymes of cellulolytic bacterial communities during ensiling of different forage sorghum varieties. **A** alpha-amylase; (**B**) Endo-1,4-beta-xylanase ; (**C**) cellulase; (**D**) beta-glucosidase; (**E**) feruloyl esterase; (**F**) alpha-glucosidase. Error bars represent the Standard deviation (SD). Different letters indicate significant differences among mean values (*p* < 0.05)

As shown in Fig. 6, the expression level of endo 1,4-beta-xylosidase (EC 3.2.1.8) in the SB3 group was significantly higher (*p* < 0.05) than that in the other groups. The expression level of feruloyl esterase (EC 3.1.1.73) in the SB1 group was significantly (*p* < 0.05) compared with that in the other treatment groups. The predicted abundance of cellulase (EC 3.2.1.4) in the SB1 group was significantly higher (*p* < 0.05) than that in all experimental groups. Similarly, the predicted abundance of beta-glucosidase (EC 3.2.1.21) in the SB1 group was also significantly higher (*p* < 0.05) than that in the other groups. In addition, the level of alpha-glucosidase (EC 3.2.1.73) in the SB4 group was significantly higher (*p* < 0.05) than that in the other treatment groups, and the level of alpha-amylase (EC 3.2.1.1) in the SB3 group was significantly higher (*p* < 0.05) than that in the other treatment groups.

## Discussion

### Analysis of agronomic traits of different forage sorghum cultivars

Differences in hay yield serve as a critical metric for evaluating the agronomic performance and environmental adaptability of forage crops [15]. This study revealed significant variations in biomass yield among five sorghum varieties, primarily attributed to their divergent adaptive capacities to the high-latitude cold environment of Hulunbuir. Comparative analysis indicated that while plant height is a major determinant of fresh forage yield, it is not the sole decisive factor. Notably, the SB4 variety exhibited both the maximum plant height and superior hay yield. This correlation suggests that SB4 possesses a robust structural framework conducive to high biomass accumulation. Increased plant height is typically associated with a well-developed vascular system and enhanced metabolic capacity, which facilitate the efficient [16]. Our findings demonstrate that these characteristics in SB4 not only promote biomass accumulation but also correlate with improved lodging resistance, thereby ensuring yield stability under the harsh climatic conditions of cold regions.

In contrast, the SB3 variety showed lower production efficiency. This disparity likely stems from limited environmental adaptability, where insufficient mechanical support and constrained assimilate transport capacity may have restricted its biomass potential [17]. These results suggest that future breeding and evaluation of sorghum varieties should prioritize the synergistic combination of culm developmental strength, plant height, and yield components, rather than focusing exclusively on height as a singular metric. For the Hulunbuir region, varieties like SB4, which demonstrate coordinated development of yield-related traits, should be prioritized for promotion. As reported by Li et al.(2025), an exclusive focus on plant height may compromise structural integrity, a premise supported by the significant negative correlation observed between plant height and stem diameter [18]. Furthermore, the positive correlation between stem diameter and panicle weight further underscores the pivotal role of robust stem development in achieving harmonious yield traits.

### Analysis of silage fermentation quality and nutritional components of different sorghum varieties

The basic principle of silage fermentation lies in the bioconversion of water-soluble carbohydrates (WSC) into organic acids, primarily LA, by lactic acid bacteria (LAB) under anaerobic conditions [19]. In this study, the SB4 cultivar exhibited the superior fermentation characteristics, marked by the most significant decline in pH and the highest accumulation of LA. These results suggest that SB4, as a fermentation substrate, possesses an efficient epiphytic LAB community, facilitating a rapid transition to homofermentative metabolism and the establishment of a stable acidic environment.

Although a low pH (< 3.80) is highly effective for preserving silage by inhibiting spoilage microorganisms, the direct feeding of excessively acidic roughage may pose a potential risk of rumen acidosis. However, in practical feeding management, this risk is effectively mitigated by formulating a balanced total mixed ration (TMR). By combining concentrates and roughage at an appropriate ratio (e.g., approximately 3:7), the acidity is adequately buffered, ensuring both nutritional balance and rumen health.

Acidification efficiency is a critical determinant in mitigating nutrient loss during the ensiling process [20]. The significantly higher CP content observed in SB4, combined with the lowest NH_3_-N levels in the high-quality group, is attributed to the rapid reduction in pH. A low-pH environment effectively inhibits the proteolytic activity of plant enzymes and blocks the metabolic processes of undesirable microorganisms, such as Clostridium and Enterobacteriaceae, thereby preventing the degradation of true protein into NH_3_-N [21]. Regarding fiber, variations in NDF and ADF contents among cultivars reflect differences in initial cell wall lignification and the extent of acid hydrolysis during fermentation.

Correlation analysis further elucidated a “microbe-metabolite-quality” nexus, where pH was significantly and negatively correlated with LA, while positively correlated with NDF and ADF. This relationship indicates that organic acid accumulation not only stabilizes the silage but also promotes partial degradation of cell wall components through acid hydrolysis and synergistic bacterial enzymatic activity. Critically, the significant negative correlation between *Lactobacillus* abundance and fiber components provides biological evidence that the dominant beneficial bacteria play a dual role: driving the rapid acidification process and enhancing the potential digestibility of the feed through fiber breakdown [22]. In conclusion, SB4 demonstrates high compatibility between its chemical composition and the required fermentative bacterial structure, making it an ideal candidate for high-quality silage production in cold regions.

Silage fermentation is a competitive process between LAB and undesirable microorganisms, which ultimately determines the bacterial community structure [23]. Given the critical impact of bacterial communities on silage quality, this study analyzed bacterial diversity and community composition using 16 S rRNA gene sequencing to elucidate the shifts occurring during the fermentation process. Significant variations in bacterial community structure were observed among different forage sorghum cultivars throughout the ensiling process.

Despite the promising results demonstrating SB4’s superiority in both agronomic traits and silage fermentation quality, it is important to note a limitation of this study. The current findings are based on a single year of field experimental data (2024). In the high-latitude cold region of Hulunbuir, inter-annual climatic variations—such as fluctuations in effective accumulative temperature, sporadic precipitation, and early frost events—can profoundly influence both crop biomass accumulation and the succession of epiphytic microbial communities. Therefore, to comprehensively validate the environmental stability and consistent performance of the SB4 cultivar, it is imperative to conduct multi-year and multi-site cultivation trials in the future. Such extended evaluations across diverse microclimates will verify its resistance to abiotic stresses and provide more robust, long-term scientific evidence for its large-scale regional adoption.

### Changes in bacterial community structure during silage process

Ensiling typically results in a reduction of bacterial alpha-diversity [24]. The Shannon index, a widely employed metric for assessing bacterial diversity, integrates both species richness and community evenness, where lower values indicate diminished diversity [25]. In the present study, cultivars SB2 and SB4 exhibited significantly lower Shannon indices compared to other hybrids. A plausible explanation is that elevated cellulase activity facilitated the degradation of structural carbohydrates in forage sorghum, thereby increasing the availability of fermentable substrates. As fermentation progressed, acid-sensitive anaerobic microorganisms were gradually eliminated, allowing beneficial taxa—specifically LAB—to establish dominance, a successional pattern consistent with previous reports. Furthermore, research by Mendez-Garcia C et al.(2015) has demonstrated that low pH serves as the primary driver for reduced bacterial diversity in acidic environments [26].

Beta-diversity analysis demonstrated significant variations in bacterial community composition across different samples, indicating that ensiling different forage sorghum varieties has a remarkable effect on the silage bacterial structure. Principal Coordinate Analysis (PCoA) revealed a distinct separation of bacterial communities during the ensiling of different varieties, suggesting that variety differences play a decisive role in shaping the silage bacterial assembling. The relatively aggregated distribution of SB2 samples is likely due to their significantly higher relative abundance of *Lactobacillus* and lower overall bacterial diversity, which further contributes to a more homogeneous community structure with reduced variation [27].

The directional succession of bacterial community structure constitutes the primary mechanism driving silage fermentation quality [28]. In this study, a taxonomic shift from Proteobacteria to Firmicutes was observed across all sorghum varieties post-ensiling, aligning with the established transition from aerobic respiration to anaerobic lactic acid fermentation in high-quality silage of Briske et al. [29]. At the genus level, SB4 exhibited the highest relative abundance of *Lactobacillus*, which corresponded with its minimum pH and maximum lactic acid concentration. This underscores the role of *Lactobacillus* as the core functional genus, which leverages water-soluble carbohydrates to rapidly acidify the environment. This process not only inhibits the proliferation of deleterious bacteria, such as Pantoea and Ralstonia, but also synergistically facilitates the degradation of NDF and ADF via acid hydrolysis, supporting the regulatory pathway of “*Lactobacillus*-driven fiber degradation and quality enhancement” of Krause et al. [30]. Conversely, the elevated abundance of Proteobacteria in SB1, coupled with higher pH and NH₃-N levels, indicates a failure to establish a stable anaerobic acidic environment, leading to protein degradation and nutrient loss mediated by spoilage microorganisms by Wang et al. [31]. Furthermore, the enrichment of Serratia in SB5 suggests potential risks to its aerobic stability. In conclusion, forage sorghum varieties determine fermentation quality by modulating bacterial succession trajectories, particularly the enrichment efficiency of *Lactobacillus.* The Firmicutes-*Lactobacillus* dominated community structure induced by SB4 represents the key microecological mechanism for achieving the synergy between high yield and superior silage quality.

The 16 S rRNA sequencing and LEfSe analysis demonstrated that the enrichment of characteristic bacterial taxa in forage sorghum silage is highly genotype-dependent. This aligns with the hypothesis of L’Hoir et al. that host genotypes shape specific silage micro-ecosystems [32]. In SB1, the significant enrichment of Hafniaceae and Hafnia-Obesumbacterium—taxa closely associated with Enterobacteriaceae Klimenko et al.—was directly linked to elevated pH and ammonia nitrogen [33]. These taxa compromise nutritional value by competing for WSC and degrading proteins, as evidenced by the low crude protein retention in SB1.Conversely, SB4 was characterized by a predominant enrichment of Lactobacillaceae and *Lactobacillus*, taxa widely identified by Wang et al. as the primary functional drivers in high-quality silage [34]. The efficient conversion of WSC into lactic acid by *Lactobacillus* rapidly lowered the pH to 3.58, effectively inhibiting spoilage bacteria such as Hafnia and Serratia while synergistically reducing the ADF content. This “High-LA, Low-fiber and Protein-preservation” profile corroborates the “Firmicutes-*Lactobacillus* dominance” theory for superior silage by Chen et al. [35]. Regarding other varieties, SB3 was characterized by the enrichment of Micrococcales and Micrococcaceae. Given that Micrococcus species can secrete cellulases and amylases of Mmango-Kaseke et al., this enrichment aligns with the high endo-1,4-beta-xylanase activity observed in SB3, facilitating hemicellulose degradation [36]. However, the lack of dominant acid-producing taxa in SB2 (Nesterenkonia) and the enrichment of Serratia in SB5—a genus linked to protein degradation and compromised aerobic stability of Zhang et al., resulted in suboptimal fermentation quality [37]. In summary, the cultivar-specific selection of bacterial taxa, particularly the *Lactobacillus* enrichment in SB4, constitutes the core micro-ecological mechanism for achieving high yield and superior quality in cold regions. These findings identify key bacterial biomarkers for selecting specialized sorghum cultivars and provide a scientific basis for tailored enzyme-microbe synergistic inoculation strategies.

The Mantel analysis (Fig. 4) elucidates the multidimensional interactive networks among nutrient composition, fermentation parameters, enzyme activities, and bacterial communities, providing a mechanistic basis for genotype-driven silage quality formation. The highly significant negative correlation between pH and LA content underscores the fundamental role of LAB in acidifying the microenvironment. This regulatory capacity was most pronounced in SB4. The significant correlations between fiber-degrading enzymes (cellulase and endo-1,4-beta-xylanase) and both LA (positive) and pH (negative) indicate that enzymatic hydrolysis not only degrades structural carbohydrates but also liberates fermentable substrates to support LAB metabolism. This synergy aligns with the “enzymatic degradation-acid production” mechanism observed by Zhang et al. [38]. At the taxonomic level, the positive correlation of *Lactobacillus* with LA and the significant association of *Ralstonia* with NH₃-N identify these genera as core functional and spoilage indicators, respectively. The “high *Lactobacillus*-low Ralstonia” community structure in SB4, coupled with optimized enzyme-microbe interactions, accounts for its superior protein preservation and fiber reduction. Conversely, the high NH₃-N and suboptimal LA in SB1 reflect a failure to establish a coordinated microbial-enzyme network. Collectively, these interactions verify the “microbe-enzyme-fermentation” regulatory axis, supporting the conclusion by Porčová et al.(2025) that cultivar-specific ecological networks are the primary determinants of silage performance in cold regions [39].

Functional profiling of the bacterial community via KEGG pathways provides insights into the metabolic potential across different forage sorghum varieties during ensiling [40]. At KEGG level 1, “Metabolism” emerged as the predominant category, accounting for 37%-39% of the total abundance. This underscores the central role of microbial-driven substrate bioconversion, which is consistent with the findings of Wang et al. in gramineous silage [41]. Within KEGG level 2, the high enrichment of “Carbohydrate Metabolism” and “Amino Acid Metabolism” highlights the primary biochemical pathways responsible for organic acid synthesis and protein preservation, as previously reported by Zheng et al. [42]. Notably, SB4—the variety with superior fermentation quality—exhibited a heightened abundance of “Carbohydrate Metabolism” pathways. This functional enrichment correlates with its maximum lactic acid concentration and minimum pH, suggesting that the SB4-associated microflora possesses a more efficient metabolic capacity for fermenting WSC into lactic acid. Conversely, the lower efficiency of protein metabolism inferred in SB1 aligns with its elevated pH and ammonia nitrogen, indicating suboptimal acidification and consequent protein degradation. While core metabolic frameworks such as nucleotide and energy metabolism remained conserved across varieties, the divergent enrichment in carbohydrate-related pathways suggests that sorghum genotypes modulate silage quality by shaping the functional traits of the bacterial community. These results support the “genotype-microbe-metabolism” regulatory hypothesis proposed by Wang et al., confirming that host-specific substrates direct the metabolic trajectory of fermentation [43].

The enzymatic profiles of the silage systems exhibited significant cultivar-specific differentiation, reflecting the regulatory role of host genotypes on carbohydrate degradation pathways during fermentation [38]. SB3 exhibited the highest activities of alpha-amylase (EC 3.2.1.1) and endo-1,4-beta-xylanase (EC 3.2.1.8). This aligns with the mechanism where amylases hydrolyze starch into fermentable sugars to support LAB and accelerate acidification. Furthermore, high xylanase activity can synergize with feruloyl esterase (EC 3.2.1.73) to cleave the ester cross-links between hemicellulose and lignin, thereby enhancing the accessibility of cellulose for degradation. This finding is consistent with the report by Campana et al., who observed that esterase-producing LAB improve fiber degradation efficiency in forage sorghum silage [44]. In contrast, SB5 showed significantly higher activities of cellulase (EC 3.2.1.4) and beta-glucosidase (EC 3.2.1.21), underscoring its superiority in the bioconversion of cellulose into soluble monosaccharides. Meanwhile, the significantly elevated alpha-glucosidase (EC 3.2.1.20) activity in SB3 and SB4 further elucidates the functional divergence in starch degradation and fiber saccharification pathways across varieties. Overall, SB3 demonstrated a dual advantage in starch and hemicellulose degradation, whereas SB5 focused on cellulose breakdown. The relatively lower enzymatic activities observed in SB1 and SB2 further confirm that the genetic background of the cultivar is a determinant of enzymatic degradation efficiency. These results provide an enzymatic basis for selecting specialized forage sorghum cultivars and suggest that tailored inoculation strategies—combining specific enzymes and microbes—could maximize the nutritional value of silage based on cultivar-specific enzymatic profiles. However, it is important to note that the functional and enzymatic profiles in this study were predicted based on 16 S rRNA gene amplicon sequencing rather than direct metagenomic or metatranscriptomic sequencing. Consequently, the current conclusions regarding metabolic pathways remain inherently predictive and descriptive. For instance, while the highly expressed alpha-glucosidase in SB4 aligns with its observed rapid pH decline (3.58), conclusively confirming a direct, causative metabolic synergy is challenging at this stage. Future studies employing targeted metagenomic and metabolomic approaches are planned to deeply investigate these dynamic metabolic mechanisms and validate the microbe-enzyme synergistic networks. This aspect will be carefully considered in future trials.

While the KEGG functional prediction based on the 16 S rRNA V3-V4 region provides a highly valuable theoretical foundation for understanding the metabolic potential of the silage microbiome, we acknowledge the inherent methodological limitations of this approach. Specifically, 16 S rRNA sequencing predicts functional potential based on taxonomic abundance rather than confirming actual gene presence or expression. Consequently, discrepancies may exist between these predictions and the true metabolic activity within the silage environment, which can only be definitively captured through direct metagenomics or metatranscriptomics. Therefore, the functional profiles presented in this study serve as a foundational step. In our upcoming follow-up research, we plan to conduct targeted metagenomic sequencing on specific treatment groups to deeply and conclusively investigate these metabolic mechanisms.

## Conclusion

SB4 is the optimal forage sorghum for Hulunbuir’s cold region, showing superior agronomic and silage traits via a *Lactobacillus*-dominated Firmicutes community, while sorghum genotypes influence bacterial structural changes and fermentation quality by regulating enzyme activity and metabolic pathways. Future research should explore psychrotolerant endophytes and breed cold-adapted cultivars and develop cultivar-specific microbe-enzyme additives to optimize silage nutritional stability in high-latitude cold areas.

## Data Availability

The raw amplicon sequencing data were deposited into the Sequence Read Archive (SRA) of NCBI database and can be accessed via accession number PRJNA1438878. Access link: https://www.ncbi.nlm.nih.gov/sra/?term=PRJNA1438878.

## Abbreviations

CP: Crude protein
DM: Dry matter
WSC: Water soluble carbohydrate
NDF: Neutral detergent fibre
ADF: Acid detergent fibre
LA: Lactic acid
AA: Acetic acid
LAB: Lactic acid bacteria
FW: Fresh weight
TN: Total nitrogen
SB1: FS3501
SB2: QCDS
SB3: F10
SB4: 3701
SB5: DJ
